# Effectiveness of immunosuppressive therapy for nephrotic syndrome in a patient with late-onset Fabry disease: a case report and literature review

**DOI:** 10.1186/s12882-019-1657-7

**Published:** 2019-12-17

**Authors:** Hironobu Fujisawa, Yosuke Nakayama, Shoichiro Nakao, Ryo Yamamoto, Yuka Kurokawa, Nao Nakamura, Akiko Nagata, Takahiro Tsukimura, Tadayasu Togawa, Hitoshi Sakuraba, Kei Fukami

**Affiliations:** 1 Division of Nephrology, Department of Medicine, Kurume University School of Medicine, 67 Asahi-machi, Kurume city, Fukuoka Japan; 2 Kirishima Kinen Hospital, Kagoshima, Japan; 3 Department of Functional Bioanalysis, Meiji Pharmaceutical University, Tokyo, Japan; 4 Department of Clinical Genetics, Meiji Pharmaceutical University, Tokyo, Japan

**Keywords:** Fabry disease, Late-onset variant, Nephrotic syndrome, Immunotherapy, Enzyme replacement therapy

## Abstract

**Background:**

Fabry disease (FD) is an X-linked lysosomal storage disorder caused by mutations of the *GLA* gene, followed by deficiency in α-galactosidase A (α-gal) activity. Nephrotic syndrome, as the renal phenotype of FD, is unusual. Here, we report the rare case of a patient with FD with nephrotic syndrome whose proteinuria disappeared by immunotherapy.

**Case presentation:**

A 67-year-old Japanese man was admitted to our hospital because of emesis, abdominal pain, and facial edema due to nephrotic syndrome. The patient was diagnosed with focal segmental glomerulosclerosis (FSGS) by renal biopsy before being diagnosed with FD, and immunotherapy was initiated. After treatment, the kidney biopsy results showed typical glycosphingolipid accumulation in the podocytes of this patient. The white blood cell α-gal activity was very low, and genetic analysis revealed a *GLA* gene variant (M296I), which is known as a late-onset genetic mutation of FD. Immunotherapy (steroids and cyclosporine A) dramatically improved the massive proteinuria. Currently, he has been undergoing enzyme replacement therapy, and his proteinuria has further decreased. There is the possibility that other nephrotic syndromes, such as minimal change nephrotic syndrome or FSGS, may co-exist in this patient.

**Conclusions:**

We experienced the rare case of a FD patient whose nephrotic syndrome disappeared by immunotherapy. These findings suggest that immunosuppressive treatment may be considered if nephrotic syndrome develops, even in patients with FD.

## Background

Fabry disease (FD) is a rare X-linked lysosomal storage disorder that is caused by a deficiency of α-galactosidase A (α-gal) activity, which leads to the accumulation of globotriaosylceramide (GB-3) in cells. The accumulation of GB-3 in various organs, such as the kidneys and heart, as well as the nervous system, has been speculated to be the mechanism involved in tissue damage [1, 2]. The major conditions associated with this disease are life-threatening complications, such as heart failure, renal failure, and cerebrovascular diseases at a young age. FD is classified into three main categories, i.e., classical variants, late-onset variants, and heterozygous variants, based on the presence or absence of characteristic symptoms, age of onset, sex, and gene mutation. The impairment of podocytes caused by GB-3 accumulation leads to the development of microalbuminuria and proteinuria as the first signs of renal functional impairment in FD [1]. However, a few FD patients with nephrotic range proteinuria have been reported, and massive proteinuria is unusual in this disease. Here, we report the rare case of a patient with late-onset FD with nephrotic syndrome, who achieved complete remission after immunosuppressive therapy, with a literature review.

## Case presentation

**Fig. 1:**
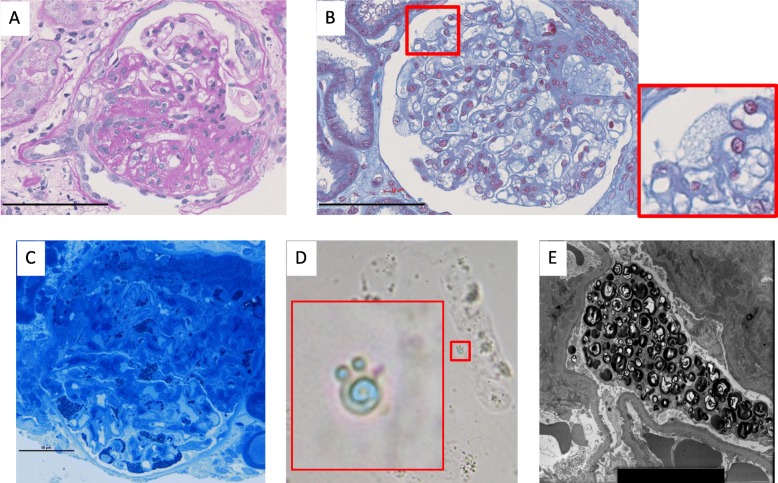
Representative images of the renal pathology in the patient. **a** Fifteen glomeruli were collected, and one showed segmental sclerosis visible on hematoxylin and eosin staining (magnification × 400, scale bar indicates 100 μm). **b** Masson trichrome staining showed vacuolization in podocytes (magnification × 400, scale bar indicates 100 μm). **c** Toluidine blue staining revealed inclusion bodies in podocytes (magnification × 400, scale bar indicates 50 μm). **d** Mulberry corpuscles were also found in the urine sediment. **e** Lamellar bodies in podocytes were observed by electron microscopy

**Fig. 2:**
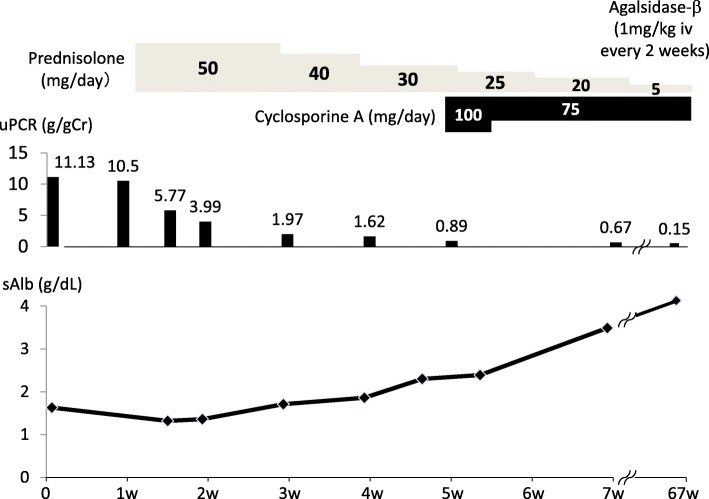
Clinical course of the patient. Before enzyme replacement therapy, a decrease in the urinary protein level was observed, and the serum albumin level was normalized with immunosuppressive therapy. uPCR = urinary protein/creatinine ratio; sAlb = serum albumin

A 67-year-old Japanese man was admitted to Munakata Suikokai General Hospital, Fukuoka, Japan, in December 2016 for angina and was subsequently diagnosed with atypical angina. He was treated with a Ca blocker (benidipine hydrochloride) and nitric acid without percutaneous coronary angiography. The urinary protein level before admission to the hospital was (±) by dipstick test, and the serum albumin level was 3.7 g/dL. His leg edema was rapidly worsening before admission to the hospital. In April 2017, he presented to the Kurume University Hospital, Fukuoka, Japan, because of emesis, abdominal pain, and facial edema due to hypoalbuminemia. The patient’s leg edema rapidly appeared 1 week before admission, suggesting the rapid onset of nephrotic syndrome. Typical FD findings, such as angiokeratoma, acroparesthesia, hypohidrosis, and corneal opacities, were absent. His mother had died from uterine body cancer at 50 years of age. Typical renal and heart disease or symptoms of FD in his brothers and maternal family members were not detected in family history. On examination, his blood pressure, pulse rate, height, and weight were 103/63 mmHg, 62 bpm (regular sinus rhythm), 167 cm, and 63 kg, respectively. Table 1 shows the patient’s laboratory data. His serum creatinine and blood urea nitrogen levels were 1.10 and 27.4 mg/dL, respectively. Urinalysis demonstrated 3+ protein as well as oval fat bodies, wide and fat casts, and mulberry cells. His urinary protein and albumin levels were 11.13 g/gCr and 1.63 g/dL, respectively. He was then diagnosed with nephrotic syndrome. After hospitalization, his creatinine level continued to increase to a peak of 2.7 mg/dL, and his urine output decreased. Because of the rapid progression of nephrotic syndrome and renal dysfunction, oral prednisolone was immediately initiated at a dose of 1 mg/kg/day. In addition, cyclosporine A (CyA) was prescribed at a dose of 100 mg/day as an additional immunosuppressive therapy. The massive proteinuria dramatically improved, and his serum creatinine and albumin levels returned to baseline (0.6 mg/dL and 4 g/dL, respectively). Percutaneous renal biopsy was performed 4 days after admission. Fifteen glomeruli were evaluated using light microscopy, and segmental sclerosis (Fig. 1a), vacuolization, and foamy changes in podocytes were observed (Fig. 1b and c). These foamy changes were also observed in the tubular epithelial cells, although to a much lesser extent than in the podocytes. These foamy cells were also found in the urine, which are known as mulberry bodies (Fig. 1d). No vascular involvement was observed. Immunohistochemistry showed no specific deposition of immunoglobulin or complement factors (data not shown). Electron microscopy revealed abundant lamellar bodies in the podocyte cytoplasm with widespread foot process fusion (Fig. 1e). These findings were compatible with renal FD. White blood cell (WBC) α-gal activity was 1 nmol/h/mg protein (normal range, 20–80 nmol/h/mg protein), and the plasma lyso-GB-3 level was 7.4 nmol/L (normal range, 0.14–0.75 nmol/L). We performed gene analysis, and the M296I mutation was detected, which is well known as a late-onset variant of FD [3]. Over the next 12 months, prednisolone and CyA were slowly tapered, and enzyme replacement therapy (ERT) (agalsidase-β, 1 mg/kg, 57 mg every 2 weeks) was initiated intravenously. His proteinuria became undetectable, and he showed sustained nephrotic syndrome remission (0.15 g/gCr) (Fig. 2). There were no adverse events related to immunotherapy or ERT during the follow-up period.

**Table 1:** Laboratory characteristics of the patient

Valuables		(normal range)
(Urinalysis)		
uPCR (g/gCr)	11.13	(< 0.15)
Selectivity Index	0.262	
Hematuria	2+	
Urine sediments		
Oval fat body	+	
Wide cast	+	
Waxy cast	+	
Fat cast	+	
Epithelial cast	+	
Mulberry cells	+	
(Biochemical examination)		
WBC (/μl)	6800	(3300–8600)
Neutrophil (%)	79.4	(40.0–71.9)
Eosinophil (%)	2.9	(0.0–5.0)
Basophil (%)	0.7	(0.0–1.0)
Lymphocyte (%)	11.4	(26.0–46.6)
Monocyte (%)	5.6	(2.3–7.7)
Red blood cell (10^4^ /μl)	496	(435–555)
Hemoglobin (g/dl)	14.9	(13.7–16.8)
Hematocrit (%)	45.3	(40.7–50.1)
Platelet (10^4^ /μl)	42.1	(15.8–34.8)
AST(U/l)	33	(13–30)
ALT(U/l)	15	(10–30)
Total protein (g/dl)	4.87	(6.6–8.1)
Serum albumin (g/dl)	1.63	(4.1–5.1)
Blood urea nitrogen (mg/dl)	27.4	(8–20)
Creatinine (mg/dl)	1.10	(0.65–1.07)
eGFR (ml/min/1.73 m^2^)	52.3	
Na (mmol/l)	143	(138–145)
K (mmol/l)	4.2	(3.6–4.8)
Cl (mmol/l)	110	(101–108)
Corrected calcium (mg/dl)	10.23	(8.8–10.1)
Phosphate (mg/dl)	3.08	(2.7–4.6)
Uric acid (mg/dl)	8.48	(3.7–7.0)
Plasma glucose (mg/dl)	105	(73–109)
Hemoglobin A1c (NGSP) (%)	5.8	(4.9–6.0)
LDL-cholesterol (mg/dl)	303.2	(65–139)
HDL-cholesterol (mg/dl)	81.3	(40–90)
Triglycerides (mg/dl)	146	(40–149)
C-reactive protein (mg/dl)	0.16	(< 0.14)
Immunoglobulin A (mg/dl)	308	(93–393)
Immunoglobulin M (mg/dl)	84	(33–138)
Immunoglobulin G (mg/dl)	827	(861–1747)
C3 (mg/dl)	131	(73–138)
C4 (mg/dl)	46	(11–31)
Anti nuclear antigen	Negative	
*HBs-Ag*	Negative	
*HCV-Ab*	Negative	
α-gal activity in WBC (nmol/h/mg protein)	1	(20–80)
Plasma Lyso-GB-3 (nmol/l)	7.4	(0.14–0.75)

eGFR was calculated using the CKD-EPI equation

*uPCR* urinary protein/creatinine ratio, *WBC* white blood cells, *AST* aspartate aminotransferase, *ALT* alanine aminotransferase, *eGFR* estimated glomerular filtration rate, *Na* sodium, *K* potassium, *Cl* chloride, *LDL* low-density lipoprotein, *HDL* high-density lipoprotein, *HBs-Ag* hepatitis B virus surface antigen, *HCV-Ab* hepatitis C virus antibody, *α-gal* alpha-galactosidase, *Lyso-GB-3* globotriaosylsphingosine

## Discussion and conclusion

Patients with FD usually show less proteinuria, at approximately 1 g/day or less, despite the accumulation of GB-3 in podocytes. Although 7.3% of male FD patients have been reported to have nephrotic-range proteinuria [4], only a few male cases of FD with nephrotic syndrome have been reported to date (Table 2). Zarate et al. reported the case of a patient with a nonsense FD mutation (W226X) with nephrotic syndrome developing secondary to minimal change disease [11]. Oral prednisolone at a dose of 2 mg/kg/day divided into two doses significantly improved his proteinuria to 100 mg/dL [11]. This team concluded that other causes of renal pathology must be considered because patients may respond to immunotherapy. Indeed, several glomerular diseases can coexist with FD, including IgA nephropathy, membranous GN, lupus nephritis, and crescentic GN, including ANCA-positive renal disease [6, 13–15]. Further, it should be noted that the rarity of proteinuria > 1 g/day in Fabry nephropathy in women should strongly suggest the presence of an alternate diagnosis. In our case, treatment with prednisolone led to remission of the heavy proteinuria. Renal pathology showed focal segmental glomerulosclerosis (FSGS), which could suggest the coexistence with FD. However, since prednisolone rapidly reduced the massive proteinuria regardless of the low selectivity index, minimal change nephrotic syndrome might not be excluded. Although we did not perform whole-exome sequencing analysis, it would be interesting to test for genes related to FSGS, although the strike rate is expected to be low in steroid-sensitive nephrotic syndrome in adults. The other mechanisms by which immunosuppressive drugs improve nephrotic syndrome in patients with FD are likely related to the inhibition of FD-associated inflammation and immune responses caused by GB-3. FD has been reported to lead a proinflammatory profile in cells, including podocytes, and immune abnormalities could be related to proteinuria and renal dysfunction in patients with FD. Indeed, increased levels of proinflammatory cytokines and oxidative stress have been reported in patients with FD, who were treated with ERT [16]. Francesco et al. reported that the proinflammatory state involves two key subsets of innate immunity and provided direct evidence of GB-3 playing a proinflammatory role, likely mediated by Toll-like receptor-4 [17]. Furthermore, compared with healthy controls, induced pluripotent stem cells from peripheral blood cell-derived endothelial cells in FD showed considerably increased reactive oxygen species (ROS) production [18]. In addition, the excess accumulation of GB-3 suppressed superoxide dismutase 2 expression and increased ROS production, finally causing vascular endothelial dysfunction in human umbilical vein endothelial cells [18]. Because prednisolone improves puromycin aminonucleoside-induced podocyte damage through the inhibition of mitochondrial dysfunction and ROS generation [19], the FD-associated massive proteinuria in our case may be improved by steroid therapy through inhibiting the dysregulation of ROS generation induced by GB-3. Moreover, the beneficial effect of calcineurin inhibitors on proteinuria is not dependent on the inhibition of nuclear factor activation in T cells but rather results from stabilization of the actin cytoskeleton in kidney podocytes [20]. Therefore, the mechanism of proteinuria normalization with a calcineurin inhibitor in this patient might be explained at least in part by stabilization of the actin cytoskeleton in podocytes. Further clinical and basic research is necessary to clarify this issue.

**Table 2:** Clinical and laboratory data of 10 cases with nephrotic syndrome in Fabry disease patients

Ref	Year	Age	Sex	Urinary protein levels	Light microscopy	Electron microscopy	Alpha-galactosidase activity	Others
Reyes Marin FA, et al. [5]	1991	22	M	7-12 g /day	Numerous vacuolated epithelial cells in the glomerular wall	Laminated bodies with myelin-like configuration	Serum 0.18 nmol/h/ml (Reference range > 12.8 nmol/h/ml)	Family history (−) *GLA* gene mutation: unknown Renal symptoms alone
Majima K, et al. [6]	1992	36	F	2-4 g /day	Diffuse membranous glomerulonephritis, and vacuolization in epithelial cells Lupus nephritis(V)	Epithelial cell cytoplasm containing osmiophilic multilamellar lipoid bodies	Leukocyte 31.3 nmol/h/mg (Reference range 21.2~53.1 nmol/h/mg) Culture skin fibroblasts 3.1 nmol/h/mg (Reference range 18.3~29.5 nmol/h/mg)	Family history (−) *GLA* gene mutation: unknown SLE (+): facial erythema (+), arthritis (+), kidney damage (+), anti-DNA antibody (+), anti-nuclear antibody (+) Urine ceramide trihexoside (+) Immunofluorescence: IgG (+), IgM (+), C3(+), C1q(+)
Thamboo TP, et al. [7]	2004	30	F	10.8 g /day	Segmental vacuolar changes in the visceral epithelial cells	Myelin-like bodies within the podocytes and tubular epithelial cells	Serum enzyme activity is normal (data not shown)	Family history (−) *GLA* gene mutation: unknown Urine ceramide trihexoside (+) Immunofluorescence: negative Response to steroid: steroid-dependent Serum Cr level: 0.47 mg/dl
Inagaki S, et al. [8]	2005	15	F	4.0 g/day	Minor glomerular abnormalities	Numerous laminated bodies in glomerular epithelial cells	Culture skin fibroblasts 68.4 nmol/h/mg (Normal subjects: 49.2 nmol/h/mg)	Family history (−) *GLA* gene mutation: unknown Urine ceramide trihexoside (+) Immunofluorescence: staining of skin fibroblasts with anti- ceramide trihexoside antibody positive
Chinen S, et al. [9]	2005	16	F	Nephrotic range	Focal segmental glomerulosclerosis	Numerous myeloid bodies in the glomerular epithelium	Leukocyte 36.1 nmol/mg P/h (Reference range: 49.8~116.4 nmol/mg P/h)	Family history (+): father, elder sister, younger sister *GLA* gene mutation: unknown Immunofluorescence: negative Response to steroid: complete remission Serum Cr level: 8.7 mg/dl (pre) 0.9 mg/dl (post steroid therapy)
Fischer EG, et al. [10]	2006	39	M	2-4 g/day	Vacuolization of the podocyte cytoplasm and variable glomerular sclerosis	Myelin-like bodies within the podocyte cytoplasm	No data	*GLA* gene mutation: unknown Immunofluorescence: negative
		73	F	3.6 g/day	Increased mesangial matrix with early nodule formation and peri-glomerular fibrosis	Myelin-like bodies within the podocyte cytoplasm	No data	*GLA* gene mutation: unknown Type II diabetes mellitus (+) Immunofluorescence: negative
Zarate YA, et al. [11]	2010	16	M	3.5 g/gCr	Prominent podocytes with a bubbly, clear, foamy cytoplasm	Abundant lamellated myelin-like inclusion in the podocyte cytoplasm Foot process fusion	Plasma 0.2 U/ml (Reference range: no information) Leukocyte 0.6 U/mg (Reference range: no information)	Family history (+) *GLA* gene mutation: W226X Immunofluorescence: negative Response to steroid: Complete remission Serum Cr level: 2.7 mg/dl (pre) 0.6 mg/dl (post steroid therapy)
Trimarchi H, et al. [12]	2013	37	M	6.8 g/day	Focal segmental glomerulosclerosis	Electron-dense laminated lipids in the cytoplasm of a podocyte	Serum or leukocyte 0.7 ng/ml (Reference range: no information)	*GLA* gene mutation: c.98A > G (D33G) Response to steroid: first therapy (Incomplete remission type I), second therapy (Incomplete remission type II)
Fujisawa H, et al.	2019	67	M	11.13 g/gCr	Segmental sclerosis, vacuolization, and foamy changes in podocytes	Abundant myelin-like inclusions in the podocyte cytoplasm	Leukocyte 1.0 nmol/h/mg protein (Reference range: 20–80 nmol/h/mg protein)	Family history (−) *GLA* gene mutation: M296I Urinary mulberry bodies (+) Plasma Lyso-GB3: 7.4 nmol/L (Reference range; 0.14–0.75 nmol/L) Immunofluorescence: negative Response to steroid: Complete remission Serum Cr level: 2.7 mg/dl (pre) 0.6 mg/dl (post steroid therapy)

*GLA* alpha-galactosidase, *SLE* systemic lupus erythematosus (Diagnostic criteria: The 1982 revised criteria for the classification of SLE), *Cr* creatinine

Although repeat renal biopsy is not available, biopsy findings on EM before and after immunosuppressive therapy and ERT would be of interest. All EM specimens showed foot process effacement, suggesting podocyte injury in this patient. It has been reported that podocyte foot process effacement is an early sign of FD without proteinuria or a decreased GFR [21]. It is speculated that foot process effacement might be recovered and lamellar bodies in podocytes might be reduced by the immunosuppressive therapy in association with the reduced proteinuria in this case.

In this case, we detected mulberry bodies in the urine. Recently, mulberry bodies and cells have become useful noninvasive diagnostic markers in patients with late-onset FD, even in those with normoalbuminuria and/or normal renal function [22]. The source of mulberry bodies is believed to be podocytes and/or distal tubular epithelial cells. Since many podocytes detach from the glomerular basement membrane in late-stage FD, mulberry bodies can easily be detected in the early stage of FD, as in this case.

This is the rare case of a patient with FD and nephrotic syndrome. Before FD was diagnosed, the patient’s urinary protein level had dramatically increased to the nephrotic range but was normalized with immunosuppressive therapy. Nephrotic syndrome is unusual in patients with FD, and few cases have been reported [5–12] (Table 2). One patient was diagnosed with FD before renal biopsy [11], whereas the others were diagnosed with FD after renal biopsy. All patients exhibited the accumulation of glycosphingolipids in the glomerular epithelial cells on renal biopsy with or without typical findings of other nephropathy. Five patients including our case received steroid therapy and 4 patients achieved complete recovery from nephrotic syndrome [7, 9, 11]. A good response to steroid therapy is atypical in FD patients with heavy proteinuria. Three nephrotic patients including our case developed renal dysfunction, which was completely improved by the immunosuppressive therapy [9, 11]. Therefore, if nephrotic-range proteinuria continues and develops, the coexistence of other nephropathies should be considered. In this regard, immunotherapy might be recommended in addition to ERT in nephrotic syndrome patients diagnosed with FD on renal biopsy.

## Data Availability

The data that support the findings of this case report are available from KF (corresponding author).

## Abbreviations

CyA: Cyclosporine A
ERT: Enzyme replacement therapy
FD: Fabry disease
FSGS: Focal segmental glomerulosclerosis
GB-3: Globotriaosylceramide
*GLA* gene: α-galactosidase A gene
ROS: Reactive oxygen species
WBC: White blood cell
α-gal activity: α-galactosidase A activity
